# News and Coming Events

**Published:** 1909-03-13

**Authors:** 


### News and Coming Events.

Dr. Walter Ramsden, M.A., M.D., Fellow and Junior Bursar of Pembroke College, has been chosen Senior Proctor for the ensuing year in the University of Oxford.

Dr. Christopher Addison, head of the anatomy department at St. Bartholomew's Hospital Medical School, has been appointed Examiner in Anatomy to the University of London, and Chairman of the Board of Intermediate Studies of the same University.

The annual conversazione of the West London Post-graduate College, Hammersmith Road, London, W., will be held on Wednesday, March 24, at 8.30 p.m. All past and present members of the College are cordially invited. Any medical men desirous of being present will have an invitation card sent on applying to me at the hospital.

At the annual meeting, on March 9, of the Liverpool Maternity Hospital and Ladies' Charity, Sir William Hartley offered to erect and equip a new hospital at a cost of £15,000. The conditions were that the committee should find a site, and that an endowment fund of £20,000, limited to fifteen years, should be raised. Sir William was thanked for his generous offer, and a contribution of £1,000 towards the endowment fund was promised by Mr. Sutton Timmis.

The Earl of Arran, who presided at the annual meeting of the Royal Westminster Ophthalmic Hospital on March 4, said that their efforts to live within their income have resulted in the refusal of awards from King Edward's Fund on the ground that they need no assistance. Fortunately, however, they have received £3,485 under the will of the late Mrs. M. H. Gill. The Chairman stated that the School of Ophthalmic Surgery continues to be most successful, and has become the recognised institution of the kind for naval surgeons.

A Blue-book issued on March 5 contains the report for 1908 of the Advisory Committee for the Tropical Diseases Research Fund. Contributions towards this fund were received from the Imperial Government and from various Colonial Governments, and it amounted in all to £3,400. Of this, £1,333 were granted to the London School of Tropical Medicine and £1,000 to the corresponding school at Liverpool; £750 to the University of London as stipend of the Professor of Proto-zoology; £100 to the University of Cambridge towards the Research Studentship in Medical Entomology; and £400 to the Australian Institute of Tropical Medicine. Reports on the official measures taken in various tropical Colonies to eradicate malaria are also included.

The National League for Physical Education and Improvement has organised a series of five lectures to be given on Thursdays at 3.30 p.m. at 35 Holland Park Avenue. Mr. Benjamin Broadbent, J.P., M.A., ex-Mayor of Huddersfield, will lecture on March 18 on "An Experiment in Baby-saving"; the Rev. H. Russell Wakefield, on March 25, "The Poor Law Report in Connection with National Health"; and Dr. Dawson Williams, on April 1, "Tuberculosis in Childhood: Its Cause and Cure." The last two lectures, whose dates are not yet fixed, will be as follows: Dr. F. E. Fremantle, "The Medical Inspection of School Children"; Professor Howard Marsh, Master of Downing College, Cambridge, "The Rôle of Health Visitors in the Scheme for Improving the Condition of the Poorer Classes."

The Lord Mayor will preside at the annual meeting of Governors of the Royal Hospital for Diseases of the Chest, City Road, to be held at the hospital on March 15 at three o'clock in the afternoon.

The Duke of Marlborough, the President, took the chair at the annual Court of Governors of the Royal National Orthopædic Hospital, held on March 4 in the hall of the new Out-patient Department in Bolsover Street, Great Portland Street. The report states that the hospital owes its bankers £3,000, and a further sum of £22,000 is required if the new hospital is to be opened free from debt.

A meeting of the medical officers and dental surgeons attached to the Metropolitan Provident Medical Association was held last week at the head office, 5 Lamb's Conduit Street. Mr. Francis Buxton, Chairman of the Association, presided, and there was a good attendance. A resolution was unanimously passed approving the action of the Association in bringing to the notice of the London County Council the work and aims of Provident Dispensaries. It was also resolved: "That in the event of the London County Council applying to the Metropolitan Provident Medical Association to undertake the medical treatment of school children through the Provident Dispensaries of London, the medical officers would heartily co-operate with the Association, and would do everything possible to meet the requirements of the educational authorities." A Medical and Dental Committee was formed to assist the Executive Committee in arranging the details of any scheme of co-operation which may be suggested between the Provident Dispensaries and the London County Council.

The Lord Mayor presided at the eighty-first annual meeting of the Royal Free Hospital held at the Mansion House on the 10th inst. The annual report states that the extraordinary expenditure (amounting to £10,533) which was incurred in 1907 for the erection and equipment of the new operating theatres and other urgently needed structural additions and improvements exhausted all the available funds, so that the year 1908 was commenced with a serious deficit, and it was necessary to borrow £5,000 from the bankers. As no funds were forthcoming to enable the committee to discharge that loan, and the interest thereon was a heavy charge, they were compelled in August to sell out a portion of the small invested funds at their disposal. Accordingly £4,982 2½ per cent. Consols were realised. The proceeds of that sale, together with the grant of £3,500 from King Edward's Hospital Fund and the payment of some legacies, enabled the committee to pay off the loan from the bankers and close the year 1908 free from debt, but with nothing in hand for the current year. During the year the total number of persons under treatment in the wards and out-patients' department was 38,476.

In the unavoidable absence of the Hon. Sydney Holland, the Mayor of Stepney (the Hon. H. L. W. Lawson) presided on March 3 at the quarterly general Court of the London Hospital. The Committee of Management report that £12,000 has been paid to the hospital as a grant from King Edward's Hospital Fund. This is the largest grant made by the Fund to any hospital. During the past year the in-patients treated numbered 14,781 and the out-patients 242,875. These figures are considerably larger than those for 1907. During the year 16,615 anæsthetics were administered, an average of 55 per day. The quinquennial appeal produced £73,038, which, in view of bad financial times, is regarded by the committee as a matter for satisfaction.

